# Protective Effects of Emodin and Chrysophanol Isolated from Marine Fungus *Aspergillus sp.* on Ethanol-Induced Toxicity in HepG2/CYP2E1 Cells

**DOI:** 10.1155/2011/452621

**Published:** 2011-09-06

**Authors:** Zhong-Ji Qian, Chen Zhang, Yong-Xin Li, Jae-Young Je, Se-Kwon Kim, Won-Kyo Jung

**Affiliations:** ^1^Department of Marine Life Science and Marine Life Research and Education Center, Chosun University, Gwangju 501-759, Republic of Korea; ^2^Department of Chemistry, Pukyong National University, Busan 608-737, Republic of Korea; ^3^Key Laboratory of Molecular Enzymology and Enzyme Engineering of Ministry Education, College of Life Science, Jilin University, Changchun 130021, China; ^4^School of Food Technology and Nutrition, Chonnam National University, Yeosu 550-749, Republic of Korea

## Abstract

Alcohol-induced liver injury progresses from fatty infiltration followed by a harmful cause of inflammation leading to an irreversible damage. In this study, two compounds (emodin and chrysophanol) isolated from marine fungus *Aspergillus sp.* were examined for their protective effects against ethanol-induced toxicity *in vitro*. Ethanol-induced HepG2/CYP2E1 cells were treated with the compounds at various concentrations, and the results showed that there was a dose-dependent decrease of gamma-glutamyl transpeptidase (GGT) activity and increase of glutathione (GSH) in the culture media with an increase in cell viability. Furthermore, the protective effects of the compounds were evaluated by protein expression levels of GGT, GSH, and CYP2E1 using Western blot. Among the compounds, emodin addressed to the ethanol-induced cytotoxicity more effectively compared to the chrysophanol. It could be suggested that emodin isolated from this genus would be a potential candidate for attenuating ethanol induced liver damage for further industrial applications such as functional food and pharmaceutical developments.

## 1. Introduction

Alcohol toxicity is one of the world's major health problems as significant numbers of people are affected due to several fatal diseases caused by alcohol [1]. Alcohol is mostly metabolized in the liver, and excessive alcohol use can lead to acute and chronic liver diseases including hepatitis, liver cirrhosis, fatty liver, and liver cancer [2]. Chronic alcohol abuse is a major health problem causing liver and pancreatic diseases and is known to impair hepatic alcohol dehydrogenase, myocardial infarction, pancreatitis, and disorders of the immune, endocrine, and reproductive systems. The diverse mechanisms are involved in the ethanol-induced hepatotoxicity while accumulating evidence shows the importance of oxidative stress mediated by reactive nitrogen species (RNS) or reactive oxygen species (ROS) [3]. Ethanol-induced oxidative stress leads to a decrease in intracellular antioxidative capacity of the liver cells including small molecular antioxidants and antioxidant enzymes such as superoxide dismutase (SOD) and glutathione (GSH). Therefore, supplementation with exogenous antioxidants has been an attractive approach to prevent or reduce ethanol-induced hepatotoxicity [4–6]. Ethanol can also be metabolized by catalase and more selectively by cytochrome P-450 2E1 (CYP2E1). Induction of CYP2E1 is proposed as a mechanism augmenting the formation of reactive paracetamol metabolites [7].

Gamma-glutamyltransferase (GGT) is a plasma membrane enzyme which catalyses extracellular glutathione (GSH); it plays a key role in the maintenance of GSH homeostasis, detoxification of xenobiotic compounds, and metabolism of endogenous biomolecules. GGT elevation indicates the involvement of GSH in metabolism since GGT facilitates GSH conjugate disposition and ensures high intracellular GSH [8]. Recent studies have shown that GGT expression is also positively regulated by iron-dependent oxidative stress. Therefore GGT appears to be a marker of oxidative stress in general [9]. 

Marine microorganisms have proven to be a rich source of biologically active natural products required for developing fine chemical agents [10]. Particularly, fungi from marine environment have shown to produce diverse secondary metabolites which are more or less similar to those produced by terrestrial fungi [11].

As a part of our ongoing studies on protective effects of metabolites from marine microorganisms on ethanol-induce toxicity, this study is focused on the metabolites isolated from a marine fungi isolated from the surface of the marine brown alga collected in the Ulsan City, Korea in 2009 and was identified as an *Aspergillus sp. *The *Aspergillus* is a ubiquitous group of filamentous fungi spanning over 200 million years of evolution. They have an impact on human health and society, and there are more than 180 officially recognized species, including 20 human pathogens as well as beneficial species used to produce foodstuffs and industrial enzymes [12].

In this study, two compounds, emodin and chrysophanol, isolated from marine fungus *Aspergillus sp.* and their chemical characteristics and protective effects on ethanol-induced toxicity in HepG2/CYP2E1 cells were investigated.

## 2. Regents

### 2.1. Materials

Dulbecco's modified Eagle's medium (DMEM), fetal bovine serum (FBS), YPG medium, dimethyl sulfoxide (DMSO), penicillin, 3-(4,5-dimethylthiazol-2-yl)-2,5-diphenyltetrazolium bromide (MTT), streptomycin, metaphosphoric acid, 2-nitro-5-thiobenzoic acid, gamma-glutamyl-p-nitroanilide, glycylglycine, naphthylethylene diamine, glutathione (GSH), *N*-acetyl-L-cysteine (NAC), 5,5′-dithiobis [2-nitrobenzoic acid] (DTNB), and nicotinamide adenine dinucleotide phosphate-oxidase (NADPH) were purchased from Gibco BRL (Grand Island, NY, USA) and Sigma-Aldrich (St Louis, MO, USA). Antibodies for GGT and GSH were obtained from Santa Cruz Biotechnology (CA), and cytochrome P450 (CYP2E1, human) was obtained from Rockland (Gilbertsville, PA). BCA protein assay kit, electrophoresis reagents, and goat antirabbit peroxidase IgG were purchased from Pierce Biotechnology Inc. (Rockford, IL). All other chemicals and solvents were of analytical grade.

### 2.2. Extraction and Isolation

The fungal strain (stock no. YL-06) was isolated from the surface of the marine brown alga collected in the Ulsan City, Korea in 2009 and identified as an *Aspergillus sp.* The fungal strain (YL-06) was stored in the 10% glycerol YPG medium at −75°C. The further culture for investigation was completed on YPG medium from 10 mL to large scale (1.0 L and 10.0 L). The fungus was cultured (30.0 L) for 30 days at 29°C in YPG medium. The culture broth and mycelium were separated, and the filtered broth was extracted with ethyl acetate to provide the broth extract (1.58 g). The broth extract extracted with ethyl acetate to provide the broth extract (1.58 g), which was fractionated by silica gel chromatography (n-5hexane/EtOAc) to generate six fractions. The further purification of the active fractions by ODS column chromatography (H_2_O in MeOH), followed by HPLC (YMC ODS-A, MeOH), yielded two compounds emodin (5.0 mg) and chrysophanol (7.0 mg).

**Emodin (1,3,8-trihydroxy-6-methylanthraquinone)**

**Figure 1:**
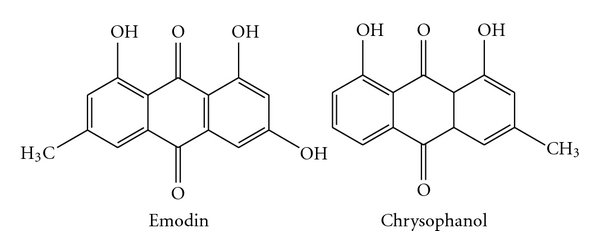
Chemical structures of emodin and chrysophanol from marine fungus *Aspergillus sp*.

Orange needles, 1H NMR (CDCl_3_) *δ* 2.37 (3H, s, 3-Me), 6.26 (1H, d, *J* = 2.45, H-5), 6.95 (1H, br s, H-2), 7.00 (1H, d, *J* = 2.45, H-7), 7.43 (1H, br s, H-4), 12.08 and 12.20 (2H, s, 1/8-OH) (Figure 1).

**Chrysophanol (1,8-dihydroxy-3-methylanthraquinone)**

Orange needles, 1H NMR (CDCl_3_, 400 MHz) *δ* (ppm) 12.03 (1H, s, OH-8), 11.93 (1H, s, OH-1), 7.72 (1H, dd, *J* = 0.76 and 7.52,H-5), 7.56 (1H, d, *J* = 8.1, H-6), 7.55 (1H, d, *J* = 0.4, H-4), 7.19 (1H, dd, *J* = 0.74 and 8.4, H-7), 7.00 (1H, d, *J* = 0.4, H-2), 2.36 (3H, s, H-3) (Figure 1).

## 3. Methods

### 3.1. Cell Culture and Viability Assay

Human hepatocellular carcinoma (HepG2) cells obtained from the American Type Culture Collection (Manassas, VA, USA) were grown in Dulbecco's modified Eagle's medium (DMEM) as described earlier containing 10% fetal bovine serum (FBS), 100 U mL^−1^ penicillin, and 100 *μ*g mL^−1^ streptomycin in a humidified atmosphere containing 5% CO_2_ and 95% air at 37°C. HepG2/CYP2E1 cell lines (HepG2 cell transfected with human CYP2E1 cDNA) were generously provided by Professor Kim from Kyunghee University Medical Center. The full length of human CYP2E1 cDNA was inserted into the *Hind*III and *Nde*I restriction sites of modified plncx (inserted *Nde*I site) expression vector (Clontech) and mapped. The HCYP2E1F-LNCX, a retroviral vector containing human CYP2E1 cDNA, was used to transfect the packaging cell line 293GPG by lipofectamine, generating a stable transfected tool to produce CYP2E1 in the virus. Virus infection of HepG2 cells was carried out by supplying a medium previously collected and filtered [13]. The cells were seeded in 24-well culture plates at a density of 1 × 10^4^ cells/mL and grown in 1 mL of growth media for 48 hr to reach 50–60% confluency and subcultured at appropriate intervals and maintained at subconfluent densities. The protein was determined using a BCA protein assay kit using bovine serum albumin as standard. All the experiments were done in triplicate.

Cytotoxic levels of the compound cultured cells were measured using MTT assay [14]. The HepG2/CYP2E1 cells were grown in 48-well plates at a density of 1 × 10^4^ cells well^−1^. After 24 h, cells were washed with fresh medium and treated with different concentrations of compound. After incubation for 48 h, the cells were rewashed and incubated with 100 *μ*L of MTT (1 mg mL^−1^) for 4 h at 37°C. Finally, 100 *μ*L of DMSO were added to solubilize the formed formazan crystals, and the amount of formazan crystal was determined by measuring the absorbance at 540 nm using a multidetection microplate reader (GENios microplate reader, Tecan Austria GmbH, Austria). The data were expressed as means of at least three independent experiments. Each value was expressed as the mean ± S.D triplicate experiments.

### 3.2. GGT Assay

GGT activity was assayed colorimetrically on a microtiter plate using gamma-glutamyl-*p*-nitroanilide as an artificial substrate. The assay is based on the GGT catalysed breakdown of artificial substrate gamma-glutamyl-*p*-nitroanilide to *p*-nitroaniline which further reacts with nitrite and naphthylethylene diamine to form a red chromogenic compound [15]. Briefly, a 20 *μ*L aliquot of conditioned cell culture media was added to 180 *μ*L of the assay mixture consisting of 185 mM Tris-Cl (pH 8.2), 2 mM gammaglutamyl-*p*-nitroanilide, 20 mM glycylglycine, 0.8 mM NaNO_2_, and 3.2 mM naphthylethylene diamine. The assay was also run using the assay mixture without the substrate, gamma-glutamyl-*p*-nitroanilide, to correct for any potential interference by the compound. After incubating the plate at 37°C for 60 min, the reaction was stopped by addition of 40 *μ*L of 1.0 N HCl. The red chromogen formation was estimated at 520 nm using a multiwell scanning spectrophotometer and corrected for the value obtained from the assay mixture devoid of the substrate.

### 3.3. Determination of Intracellular GSH Contents

The cells were ruptured in 100 *μ*L of 5% metaphosphoric acid and centrifuged at 10,000 ×g for 15 min to obtain clear supernatants. The supernatants were used for the determination of total GSH by the enzymatic recycling method using DTNB [16]. The assay was carried out on a microtiter plate. The reaction mixture (100 *μ*L) consisted of 143 mM NaPO4 (pH 7.5), 6.3 mM EDTA, 0.6 mM DTNB, 0.25 mM NADPH, 0.25 U mL^−1^ GSH reductase, and 2.0 *μ*L compound. The plate was incubated at 37°C for 60 min, and the formation of 2-nitro-5-thiobenzoic acid was monitored by absorbance at 415 nm and converted to GSH concentration using a calibration curve with known amounts of GSH.

### 3.4. Western Blot Analysis

Western blotting was performed according to standard procedures. Briefly, cells were cultured at a density of 1 × 10^4^ cells mL^−1^ in 6-well plate culture dishes with serum-free medium. After incubation for 48 h, the cells were treated with different concentrations of compound for 1 h and then treated with 1.0 M ethanol for 48 h with serum-free medium. Cells were lysed in lysis buffer containing 50 mM Tris-HCl (pH 8.0), 0.4% Nonidet P-40, 120 mM NaCl, 1.5 mM MgCl_2_, 2 mM phenylmethylsulfonyl fluoride, 80 *μ*g mL^−1^ leupeptin, 3 mM NaF, and 1 mM DTT at 4°C for 30 min. Total protein was extracted, and 100 *μ*g mL^−1^ of protein were separated using a 10% SDS-polyacrylamide gel and 5% stacking gels and transferred onto a nitrocellulose membrane (Amersham Pharmacia Biotech., England, UK). The membrane was blocked for 1.5 h at 37°C using TBS-T buffer containing 0.1% Tween-20 and 3% BSA. After washing the membrane with TBS-T twice, the blots were incubated for 1 hr with suitable antibodies at 25°C. The respective proteins were detected with a chemiluminescent ECL assay kit (Amersham Pharmacia Biosciences, NJ, USA) according to the manufacturer's instructions. The western blot bands were visualized using a LAS-3000 system and quantified by Multi-Gauge V3.0 software (Fujifilm Life Science, Tokyo, Japan).

### 3.5. Statistical Analysis

Each value was expressed as means ± S.E.M (*n* = 3). The statistical significance of differences was analyzed by Student's *t*-test using SPSS (Chicago, IL, USA).

## 4. Results

### 4.1. Cytotoxic Effects of Ethanol and Compound (Emodin and Chrysophanol on HepG2/CYP2E1 Cells and Ethanol Induced HepG2/CYP2E1 Cells)

**Figure 2:**
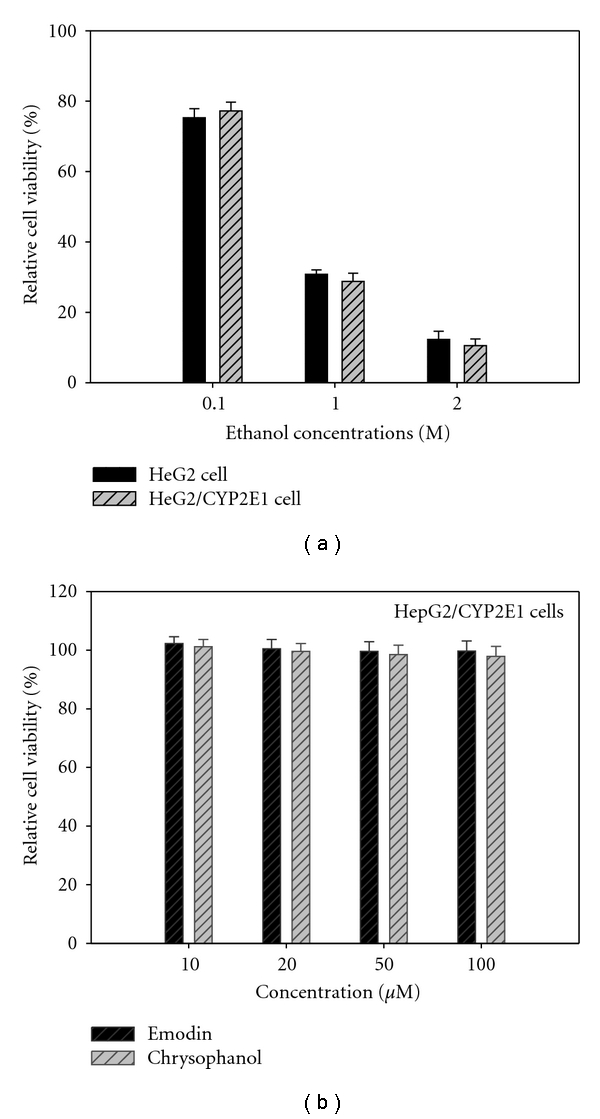
(a) Cytocompatibility of different concentrations (0.1–2 M) of ethanol on HeG2 and HeG2/CYP2E1 cells. (b) Cytocompatibility of emodin and chrysophanol on HepG2/CYP2E1 cells. Different concentrations of compounds were applied to the cells for 48 h, and cell viability was assessed by MTT assay as described in the text. Results are means ± standard error of three independent experiments.

The study first examined the cytotoxicity of different concentrations of ethanol on HeG2 and HeG2/CYP2E1 cell lines. The cells were treated with 0.1–2.0 M ethanol for 48 h and subjected to MTT assay to assess cell viability. As shown in Figure 2(a), the cells appeared to be quite resistant to ethanol up to 0.1 M but 2.0 M ethanol induced a severe loss of cell viability. Therefore, based on this viability data, the tested concentration of ethanol was 1.0 M, and HepG2/CYP2E1 cell lines can be transfected with human CYP2E1 cDNA; so we selected HeG2/CYP2E1 cell line for further experiments. The cytotoxic effects of emodin and chrysophanol on HepG2/CYP2E1 cells were determined by MTT assay. As shown in Figure 2(b), both emodin and chrysophanol exhibited no significant effects of cell proliferation at the tested concentrations (10–100 *μ*M) after treatment for 48 h. Based on this viability data, the tested concentrations of emodin and chrysophanol were selected in the range of 10–100 *μ*M for investigating the protective effects on ethanol-induced cytotoxicity. 

**Figure 3:**
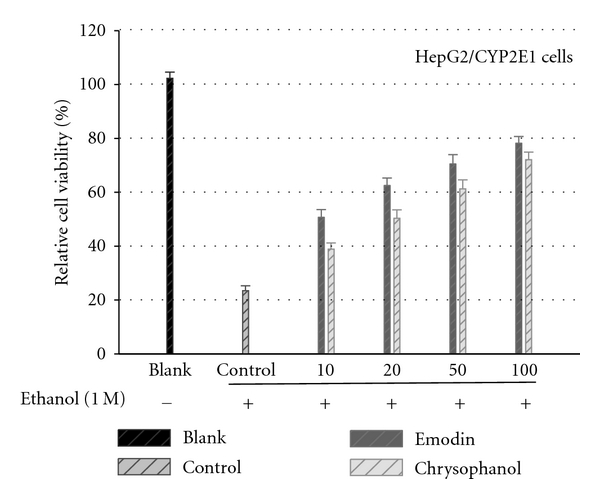
Effects of emodin and chrysophanol on the ethanol-induced cytotoxicity. The HepG2/CYP2E1 cells pretreated with compound at various concentrations for 1 h prior to the treatment with 1.0 M ethanol for 48 h. The cells were then subjected to cell viability test. Results are means  ± standard error of three independent experiments.

The HepG2/CYP2E1 cells were pretreated with compound at various concentrations (10–100 *μ*M) for 1 h prior to the treatment with 1.0 M ethanol for 48 h. The cells were then subjected to cell viability test. As expected (Figure 3), ethanol treat cell death (23.5% cell survival); however, the effect was almost completely abrogated when the cells were cotreated with emodin, chrysophanol together with ethanol (1.0 M) (emodin, 78.2% at 100 *μ*M; chrysophanol, 72.1% at 100 *μ*M cell survival), suggesting that emodin and chrysophanol have protective effect against ethanol-induced cytotoxicity in HepG2/CYP2E1 cells.

### 4.2. Effect of Emodin and Chrysophanol on Ethanol-Induced GGT and GSH Depletion in HepG2/CYP2E1 Cells

**Figure 4:**
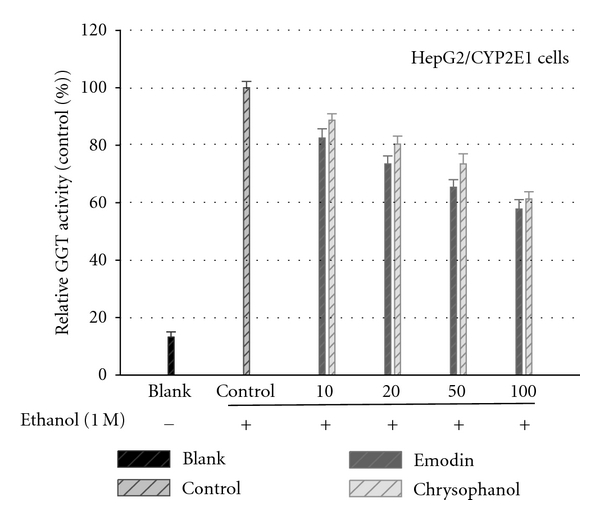
Constituents on the ethanol-induced GGT release. The HepG2/CYP2E1 cells pretreated with emodin and chrysophanol at various concentrations for 1 h prior to the treatment with 1.0 M ethanol for 48 h. The culture media were used for GGT assay. Results are means ± standard error of three independent experiments.

Serum gamma-glutamyltransferase (GGT) appeared to attenuate the ethanol-induced cytotoxicity; therefore the potential influence of emodin and chrysophanol on GGT activity was examined. As shown in Figure 4, ethanol (1.0 M) treatment of the cells increased GGT activity in the culture media of HepG2/CYP2E1 cell. Both emodin and chrysophanol inhibited the GGT increase dose-dependent manner (10–100 *μ*M) after 48 h.

**Figure 5:**
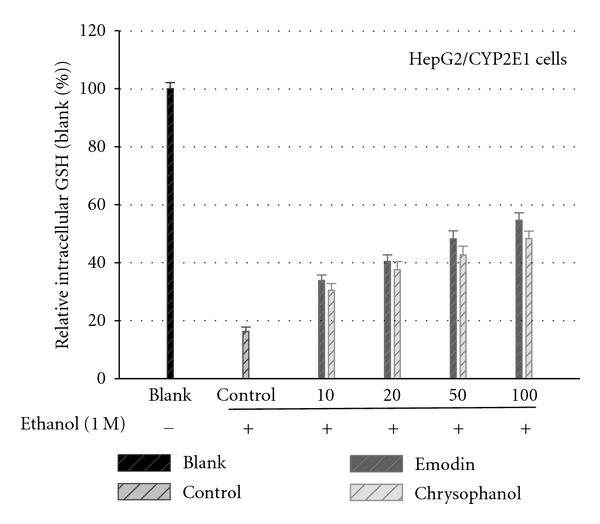
Effects of emodin and chrysophanol on the intracellular glutathione contents in the absence and presence of ethanol treatments. The HepG2/CYP2E1 cells pretreated with emodin and chrysophanol at various concentrations for 1 h prior to the treatment with 1.0 M ethanol for 48 h. The cell lysates in 5% metaphosphoric acid were used for the determination of intracellular GSH content for 48 h. Results are means ± standard error of three independent experiments.

Cellular GSH levels at different concentrations (10–100 *μ*M) of emodin and chrysophanol are shown in Figure 5. At the high concentration of the compound the GSH levels were significantly higher than those at the low concentration of the compound. Parallel to GGT activity results, both compounds (emodin and chrysophanol) exhibited elevation of GSH activity in dose dependent manner after 48 h.

### 4.3. Effects on GGT, GSH, and CYP2E1 Protein Expression of Emodin and Chrysophanol Evaluated by Western Blot

**Figure 6:**
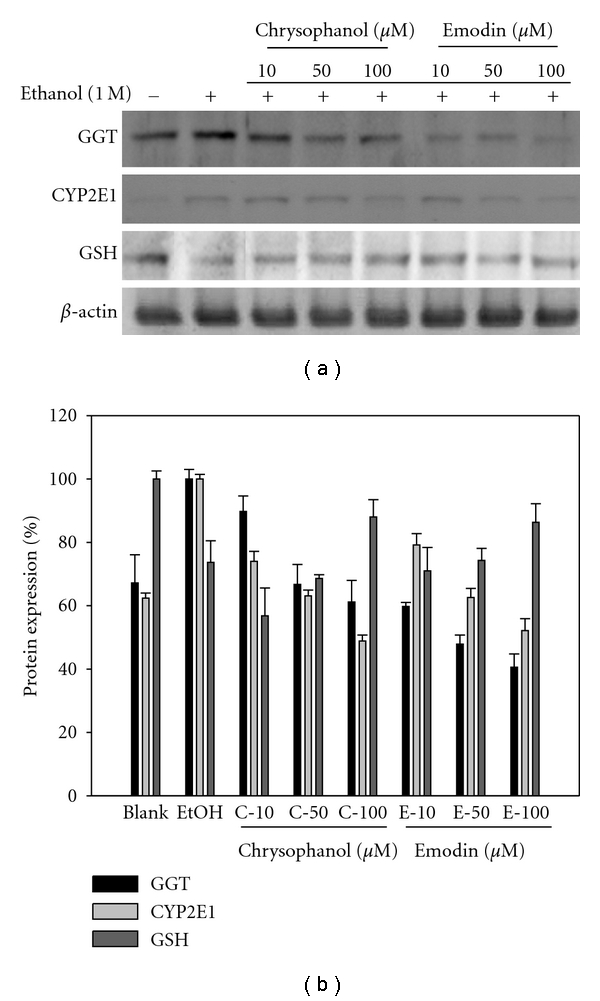
Effects of emodin and chrysophanol on the protein levels of GGT, GSH, and CYP2E in ethanol-induced HepG2/CYP2E1. Cells were treated with the compounds at different concentrations (10, 50, and 100 *μ*M) and compared with ethanol nontreated group. The expression levels of protein were determined using Western blot analysis.

Besides the activity assays, levels of GGT, GSH, and CYP2E1 proteins were also examined by Western blot analysis after treatment with emodin and chrysophanol on ethanol-induced HepG2/CYP2E1 cells. Compounds treatment decreased the ethanol-induced elevated GGT and CYP2E1 protein levels in a dose-dependent manner. And both of the compounds increased the GSH protein levels dose-dependently against ethanol-induced depletion (Figure 6).

## 5. Discussion

Human hepatocellular carcinoma (HepG2) cells are known to metabolize ethanol nonoxidatively to fatty acid ethyl esters (FAEEs) [17]. Also, due to their many genotypic and phenotypic similarities to human hepatocytes, HepG2 cells are being often used for a variety of drug metabolism and toxicity studies, such as hepatic alcohol dehydrogenase (ADH) and CYP2E1 [18]. Previous studies have demonstrated that CYP2E1 was detectable in HepG2/CYP2E1 cells and HepG2 cells were not detectable. Consequently, our used HepG2 cells transfected with CYP2E1 in the present study to understand the metabolic basis of ethanol-induced hepatocellular injury. Western blot analysis clearly demonstrates over-expression of CYP2E1 in HepG2/CYP2E1 cells and HepG2 cell was not detectable (data not shown). 

Ethanol consumption is known to increase the serum GGT level by inducing its expression, although it is also evident that alcohol-induced cellular damage leads to the enzyme release from the membranes. Many factors other than alcohol intake are associated with increased levels of GGT, in particular body mass index, diabetes mellitus, and serum total cholesterol [19]. GGT is one of the longest established biochemical tests for excessive alcohol consumption. Even though GGT is widely used as a marker of liver damage or alcohol abuse in clinics, its role in the ethanol toxicity has been elusive [20]. It is possibly involved in reabsorption of glutathione from the glomerular filtrate and protection against oxidative stress, via maintenance of intracellular glutathione levels [21]. GGT levels increase in response to exposure to a variety of drugs and alcohol. This suggests that GGT inhibition can provide positive effects on cell survival under stressed conditions. Therefore, the protective effects of emodin and chrysophanol could be attributed at least partly to its inhibition of GGT activity that appears to play a critical role in the ethanol toxicity. GGT catalyses extracellular GSH breakdown and produces the metabolites used for GSH synthesis inside the cells. Therefore, GGT activity might be in great demand if intracellular GSH is depleted by ethanol toxicity [22]. However, GGT expression or activation may not lead to a restoration of intracellular GSH because the reaction products of GGT could potentially induce oxidative stress [23]. 

 GSH, the most abundant antioxidant in cells, plays a major role in the defense against oxidative stress-induced cellular injury and is essential for the maintenance of intracellular redox balance [2]. Neuman et al. [24] reported a dramatic decrease in mitochondrial GSH in isolated hepatocytes exposed to alcohol. Ethanol increased ROS production, decreased GSH, and increased lipid peroxidation [25]. Incubation of HepG2 cells with ethanol induced oxidative stress and leaves the cells vulnerable to further injury by ROS. The results showed that the levels of GSH in HepG2 cells exposed to ethanol were significantly lower than GSH levels in nontreated cells. And also the data showed that the GSH levels decreased in ethanol-induced HepG2 cells while it increased in compounds (emodin and chrysophanol) treated cells. A dose-dependent GSH elevation was observed in compounds-treated cells after 48 h exposure to ethanol. Therefore, it is evident that compound treatment can relieve ethanol-induced cellular injury in HepG2/CYP2E1 cells which causes imbalance of cellular antioxidative system due to incubation with ethanol. The cytoprotective effects of emodin and chrysophanol are thus likely to be associated with these enzymes.

CYP2E1 could be induced by a broad variety of chemicals, such as ethanol. Ethanol increases the activity of cytochrome P450 2E1 (CYP2E1), which can metabolize alcohol and generate ROS [26, 27]. The CYP2E1 constitutes the microsomal ethanol oxidizing system, which is inducible by higher amounts of ethanol and other xenobiotics. Ethanol can also be metabolized by catalase and more selectively by cytochrome P-450 2E1 (CYP2E1) [28]. Acetaldehyde is the first oxidation product of ethanol. Due to high reactivity it is responsible for many aspects of alcohol-related liver injury. In a recent study CYP2E1 activity, cellular GSH and GGT levels were chosen to assess hepatocyte damage caused by ethanol exposure [29]. Due to the complicated nature of ethanol toxicity, various strategies have been suggested to protect cells from it. They include inhibition of ethanol metabolism leading to the production of toxic metabolites such as acetaldehyde and inhibition of ROS production from nitric oxide synthases, NADPH oxidase, and mitochondrial electron transport [4]. GGT catalyses extracellular GSH breakdown and produces the metabolites used for GSH synthesis inside the cells. Thus GGT activity might be in great demand if intracellular GSH is depleted by ethanol toxicity.

Recently, marine microorganisms have been well known as an important source to produce naturally bioactive secondary metabolites including phenols and polyphenols with unique linkages (ether and/or phenyl). In our results, emodin attenuated the ethanol cytotoxicity effectively compared to the chrysophanol. The active focus of emodin and chrysophanol components is ascribed to the phenolic hydroxyl groups attached to the ring structure. The structural diversity in each compound is determined by the number and the arrangement of hydroxyl groups and by the methyl groups. They were found to rule the biological effect of such systems. Therefore it could be suggested that structure-activity relationships (SARs) mainly depend on the number of phenolic ring substituents and phenyl ether linkages. The protecting effect of emodin with three hydroxyl groups was stronger than that of chrysophanol containing two hydroxyl group-substituted compounds on ethanol-induce cytotoxicity. Emodin is a natural anthraqinone compound, that is, an active component of the dried root of Rhei Rhizoma (Rheum palmatum, Daehwang in Korean) and is also present in many herbs and vegetables including cabbage, lettuce, beans, and peas [30]. Emodin is known to have immunosuppressive effect, hepatoprotective effect, antiinflammatory, antimicrobial, antiviral, anticancer and wound healing properties [31]. It could be suggested that emodin from this genus would be more potential candidate for attenuating ethanol-induced liver damage for further industrial applications such as in functional foods and pharmaceuticals.

## 6. Conclusion

In conclusion, our study demonstrated that emodin and chrysophanol isolated from marine fungus *Aspergillus sp. *are attenuating the ethanol-induced cytotoxicity of HepG2/CYP2E1 cells. Moreover, the ethanol-induced cytotoxicity could be attenuated by inhibition of GGT activity and CYP2E1 protein expression and providing increased levels of intracellular GSH. Therefore, it suggests that emodin and chrysophanol may provide a novel strategy for attenuating ethanol-induced liver damage.
